# The Effect of a Web-Based Cervical Cancer Survivor’s Story on Parents' Behavior and Willingness to Consider Human Papillomavirus Vaccination for Daughters: Randomized Controlled Trial

**DOI:** 10.2196/34715

**Published:** 2022-05-25

**Authors:** Yukio Suzuki, Akiko Sukegawa, Yutaka Ueda, Masayuki Sekine, Takayuki Enomoto, Alexander Melamed, Jason D Wright, Etsuko Miyagi

**Affiliations:** 1 Department of Obstetrics and Gynecology Yokohama City University Graduate School of Medicine Yokohama Japan; 2 Division of Gynecologic Oncology Department of Obstetrics and Gynecology Columbia University Vagelos College of Physicians and Surgeons New York, NY United States; 3 Department of Obstetrics and Gynecology Osaka University Graduate School of Medicine Suita Japan; 4 Department of Obstetrics and Gynecology Niigata University Graduate School of Medical and Dental Sciences Niigata Japan

**Keywords:** human papilloma virus vaccination, vaccination, vaccine, vaccine hesitancy, cancer survivor, narrative story, web based, randomized controlled trial, RCT, HPV, human papilloma virus, virus, hesitancy, cancer, willingness, behavior, parent

## Abstract

**Background:**

Providing adequate information to parents who have children eligible for human papillomavirus (HPV) vaccination is essential to overcoming vaccine hesitancy in Japan, where the government recommendation has been suspended. However, prior trials assessing the effect of brief educational tools have shown only limited effects on increasing the willingness of parents to vaccinate their daughters.

**Objective:**

The aim of this trial is to assess the effect of a cervical cancer survivor’s story on the willingness of parents to get HPV vaccination for their daughters.

**Methods:**

In this double-blinded, randomized controlled trial (RCT) implemented online, we enrolled 2175 participants aged 30-59 years in March 2020 via a webpage and provided them with a questionnaire related to the following aspects: awareness regarding HPV infection and HPV vaccination, and willingness for HPV vaccination. Participants were randomly assigned (1:1) to see a short film on a cervical cancer survivor or nothing, stratified by sex (male vs female) and willingness for HPV vaccination prior to randomization (yes vs no). The primary endpoint was the rate of parents who agreed for HPV vaccination for their daughters. The secondary endpoint was the rate of parents who agreed for HPV vaccination for their daughters and the HPV vaccination rate at 3 months. The risk ratio (RR) was used to assess the interventional effect.

**Results:**

Of 2175 participants, 1266 (58.2%) were men and 909 (41.8%) were women. A total of 191 (8.8%) participants were willing to consider HPV vaccination prior to randomization. Only 339 (15.6%) participants were aware of the benefits of HPV vaccination. In contrast, 562 (25.8%) participants were aware of the adverse events of HPV vaccination. Although only 476 (21.9%) of the respondents displayed a willingness to vaccinate their daughters for HPV, there were 7.5% more respondents in the intervention group with this willingness immediately after watching the short film (RR 1.41, 95% CI 1.20-1.66). In a subanalysis, the willingness in males to vaccinate daughters was significantly higher in the intervention group (RR 1.50, 95% CI 1.25-1.81); however, such a difference was not observed among females (RR 1.21, 95% CI 0.88-1.66). In the follow-up survey at 3 months, 1807 (83.1%) participants responded. Of these, 149 (8.2%) responded that they had had their daughters receive vaccination during the 3 months, even though we could not see the effect of the intervention: 77 (7.9%) in the intervention group and 72 (8.7%) in the control group.

**Conclusions:**

A cervical cancer survivor’s story increases immediate willingness to consider HPV vaccination, but the effect does not last for 3 months. Furthermore, this narrative approach to parents does not increase vaccination rates in children eligible for HPV vaccination.

**Trial Registration:**

UMIN Clinical Trials Registry UMIN000039273; https://tinyurl.com/bdzjp4yf

## Introduction

### Background

To eliminate cervical cancer, the World Health Organization (WHO) set a future goal that 90% of girls worldwide would be vaccinated for the human papillomavirus (HPV) by the age of 15 years, by the year 2030 [1]. In fact, a number of countries with high vaccination coverage have already achieved an immunization rate of 90% or higher in this target population [1]. In Japan, due to the suspension of proactive government recommendations since 2013, most people have been hesitant to get the HPV vaccination [2-4]. As a result, the vaccination rate of the target population has been estimated to be below 1% [5,6]. The incidence of HPV vaccination has been changing in recent years, with quadruple the number of HPV vaccination doses supplied in the first 3 months of 2021 compared to the same period 4 years ago [7]. Although the trend in the HPV vaccination rate seems to have been increasing in Japan, it is still important to find better ways to promote HPV vaccination—not only in Japan but also in other countries that have not accomplished the 90% vaccination goal. Furthermore, given that the nationwide proactive recommendation of HPV vaccination for girls aged 11-16 years resumed in April 2022 in Japan [8], it should be necessary to find effective ways to promote the vaccination rate.

In many countries, vaccine hesitancy is a crucial public health issue that governments continue to struggle with [9,10]. Vaccine hesitancy is usually based on perceived safety concerns associated with receiving the vaccines. In Japan, the repeated broadcast of adverse events, which are regarded as functional disorders, has discouraged many individuals from getting their daughters vaccinated [2,5,11]. In the United States, the HPV vaccination rate has risen to around 50% among vaccine-eligible adolescents, with vaccine hesitancy as a main source of the ongoing problem [12]. It has been demonstrated that the most frequent reason for hesitancy stems from people’s concerns regarding safety and adverse effects [12]. As social media continues to become a major source for public health information, it has become increasingly difficult to filter out wrong and inaccurate information.

Providing brief scientific information regarding HPV vaccination through websites has been shown to be an effective way of disseminating the importance of vaccination [4], with the potential to change people’s sentiments toward vaccination for their children. Our previous randomized controlled trial (RCT) showed that the brief education material significantly increased the number of people willing to consider HPV vaccination for their children; however, the effect was seen only in the men’s cohort [4]. The reason for this was thought to be that more negative attitudes toward HPV vaccination existed among women compared to men, which could then affect women’s attitudes. The difference in the effectiveness of educational interventions between sexes was a notable outcome. Therefore, there is a need to assess whether other educational approaches can change women’s willingness to consider HPV vaccination for their daughters.

### Goal of the Study

The aim of this study is to assess whether a cervical cancer survivor’s story could change parents’ minds about HPV vaccination. We plan to build up the theory of a better way to change people’s minds and behaviors to overcome vaccine hesitancy. Little is known about evidence-based interventions promoting the prevention of HPV, so this trial could provide a novel insight into digital educational methods for promoting disease prevention through vaccination to the general public.

## Methods

### Study Design and Participants

**Figure 1:**
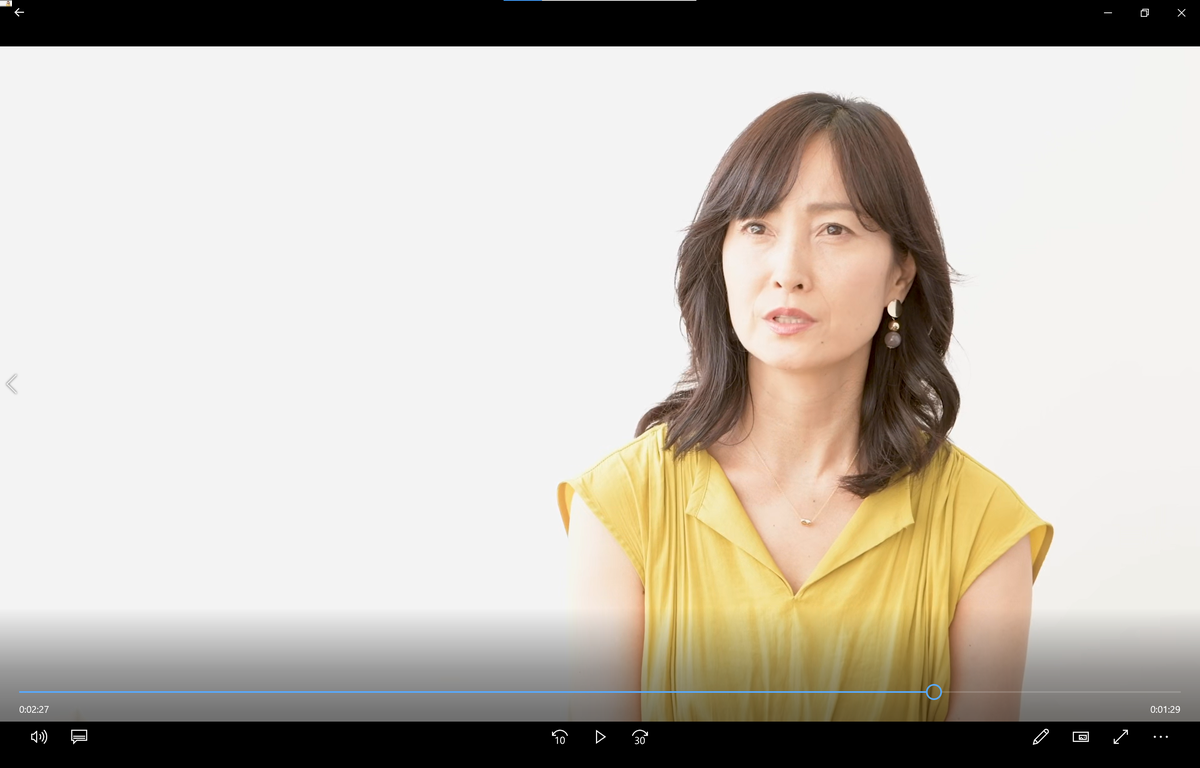
Short film on Yoko Matsuda, a cervical cancer survivor.

A total of 2175 participants were recruited in March 2020 via a webpage dedicated to this trial. These were registered members of the research panel owned by NTT Com Online Marketing Solutions Corporation (Tokyo, Japan) and used for consumer satisfaction, marketing, and academic research [13]. More than 53,000 people in Japan have registered on this research panel based on their applications [14]. The eligible participants were aged between 30 and 59 years and had at least 1 daughter between sixth-grade elementary (11 and 12 years of age) and third-grade high school (17 and 18 years of age). The parents who had their daughters vaccinated against HPV were not allowed to join this study. The first survey was conducted on March 19-30, 2020, while the 3-month follow-up survey was conducted on June 26-July 6, 2020. Participants were recruited until the target sample size was reached after considering a dropout rate of approximately 30%-40% for the 3-month follow-up. Each participant responded to an identical willingness questionnaire. However, only participants in the intervention group watched a short film on a cancer survivor (as displayed in Figure 1 and Multimedia Appendix 1) prior to taking the survey; participants who were assigned to the control group could choose to watch the short film after all questionnaires, including a follow-up questionnaire, were completed. The full process of this trial was implemented online. The primary endpoint was the rate of parents who have willingness to vaccinate their daughters against HPV. The secondary endpoint was the HPV vaccination rate at a 3-month follow-up and awareness regarding the prevention of cervical cancer.

### Information Prior to Randomization

Basic information about the benefits and adverse effects of HPV vaccination was impartially provided to all participants on a 2-slide handout prior to randomization, with the aim of sharing scientific nonnarrative information. On the first slide, the perceptions of the Japanese Ministry of Health, Labour and Welfare (MHLW) regarding adverse effects after receiving HPV vaccination was provided [3]. The following 3 points were shared:

- “Most vaccinated people experience pain and swelling on the arm where the shot is given.”
- “Severe adverse effects, such as unspecified body pain and sudden loss of strength, are rarely reported and are thought to be due to a functional disorder with unknown specific cause.”
- “Healthcare providers can access information on the website of MHLW regarding individuals who received the shot and developed continuous adverse effects.”

On the second slide, 3 points regarding cervical cancer and HPV vaccination were provided:

- “In Japan, approximately 10,000 people a year are diagnosed with cervical cancer, while about 3,000 people die annually.”
- “Many developed countries recommend HPV vaccination in adolescence as a national prevention program.”
- “The HPV vaccine, which the World Health Organization (WHO) reports to be safe, can lead to the prevention of 70% of cervical cancers and other HPV-related cancers (e.g., oropharyngeal cancer, oral cancer, vulval cancer, and anal cancer).”

### Intervention

In this study, a 4-minute-long short film was used as the intervention. In this film, a cervical cancer survivor who had undergone radical hysterectomy with bilateral salpingo-oophorectomy and pelvic lymphadenectomy talked about her experience—from the beginning of the diagnosis to the sequelae of first-line therapy. She was a singer, songwriter, and actress in Japan. Seven messages were inserted as subtitles throughout her talk. The added subtitles were as follows:

- “She was diagnosed with cervical cancer at 31 years old.”
- “In Japan, approximately 10,000 people a year are diagnosed with cervical cancer and about 3,000 people die annually.”
- “Undergoing surgery wasn’t the end of suffering.”
- “The burden of cervical cancer was greater than expected and is relatively seen in younger ages.”
- “Cervical cancer can be prevented by HPV vaccination and Pap smears.”
- “A lot of children have lost their chance to prevent cervical cancer in Japan.”
- “Why don’t we take action for our children’s future?”

We did not use any psychosocial theoretical frameworks to manipulate the participants’ mindset or behavior. The short film was produced by Ideas and Effects, Ltd., and supervised by the first author (YS) and the last author (EM), for use in educational campaigns.

### Randomization

Participants were randomly assigned (1:1) to each group using a web-based randomization procedure. The randomization with minimization was stratified by sex (female/mother and male/father) and willingness toward HPV vaccination prior to randomization (yes or no). Randomization was performed using the web research system of the NTT Data Institute of Management Consulting, Inc. The participants and investigators were double-blinded to the study distribution. Once the upper limit of each stratum was reached, new participants could not be added to the web system. This ensured a uniform distribution of stratification factors. In the intervention group, we provided the narrative short film prior to answering questions related to the willingness to prevent HPV, following consent for the online study.

### Questionnaire

The participant demographics included sex (male/father and female/mother), age group (thirties, forties, fifties), willingness to use HPV vaccination prior to randomization (yes or no), marital status, number of children, education, household income, and tobacco use. The individual background information already existed in the research panel database prior to our study, except for the level of willingness to use the HPV vaccine. Information regarding the HPV vaccination history of respondents and a previous Papanicolaou (Pap) test for female participants was also collected for this study. In total, data of marital status, household income, and education level were collected from 1550 participants.

The participants completed a 7-item awareness questionnaire to determine HPV awareness as background information. They were instructed to answer either “Yes, I have heard of it” or “I haven’t heard of it” for each question. We defined those who answered “I haven’t heard of it” for all questions as the no-awareness group, whereas those who answered “Yes, I have heard of it” for at least 1 question were defined as the normal-awareness group.

The awareness questions (AQs) were as follows:

- AQ 1. It is possible to detect both cancer and precancerous lesions through cervical cancer screening.
- AQ 2. Sexual experience is associated with HPV infection.
- AQ 3. Cervical cancer screening is necessary for women even after vaccination.
- AQ 4. I have heard of the benefits of the HPV vaccine.
- AQ 5. I have heard of the adverse events associated with the HPV vaccine.
- AQ 6. HPV can cause anal cancer and oropharyngeal cancer in males, other than cervical cancer in women.
- AQ 7. HPV vaccination is included in the national immunization program (routine vaccination schedule) and publicly funded for children from sixth-grade elementary school students to first-grade high school students (12-16 years of age). However, the MHLW suspended a proactive recommendation for HPV vaccination due to the suspicious relationship between the vaccine and unspecific chronic pain.

The willingness questions (WQs) after the intervention were as follows:

- WQ 1. Would you consider getting your daughter vaccinated against HPV? (Yes/No/I’m not sure)
- WQ 2. Would you consider undergoing a Pap smear? If male, would you want your family member or partner to undergo a smear test? (Yes/No/I’m not sure)
- WQ 3. Do you think the HPV vaccination program should be actively recommended by the government? (Yes/No/I’m not sure)
- WQ 4. Do you plan to inform family members, friends, or others about cervical cancer prevention and screening (through Instagram, Facebook, LINE, Twitter, TikTok, etc)? (Yes/No/I’m not sure)
- WQ 5. Are you going to make an appointment for HPV vaccination for your daughter as soon as possible? (Yes/No)

The follow-up questions (FQs) after 3 months were as follows:

- FQ 1. Has your daughter been vaccinated against HPV after the first-round questionnaire? (Yes/Only an appointment/No)

Participants who answered “No” were subsequently tasked to answer the following items:

- FQ 1’. Would you consider getting your daughter vaccinated against HPV? (Yes/No/I’m not sure)

FQs 2-5 were identical to WQs 2-5.

Participants who answered “Yes” or “Only an appointment” in FQ 1 were tasked to answered only FQs 2-4.

### Statistical Analysis

The chi-square (*χ*^2^) test, Student *t* test, and risk ratio (RR) were used for statistical analyses of background characteristics, baseline knowledge level, and primary/secondary outcomes. The background characteristics and baseline knowledge between both groups were not significantly different, so we did not adjust the present variables. All statistical analyses were performed using SPSS Statistics version 28 (IBM). The sample size was calculated as 80% power to detect a 10% effect in the intervention group (increased from 25% in the control group to 35% in the intervention group) with a 2-sided *P* value of .05. Statistical significance was set as less than .05. The hypothetical baseline willingness rate was determined based on our previous studies; 23.6% of participants who were provided with information regarding the benefit of HPV vaccination considered getting their daughter vaccinated [4]. The sample size was calculated as 784 (392 vs 392), and the effect of the intervention estimated a 10% increase after 3 months. We set the target number of participants recruited at 3 times the calculated sample size because the web-research company estimated the follow-up rate to be 30%-40% at 3 months after the initial survey.

### Ethical Considerations and Funding

The trial protocol was approved by the institutional research ethics committee of the Yokohama City University School of Medicine (B200109003). The trial registration number was UMIN000039273. We received research funding from the Japan Agency for Medical Research and Development (Grant 19ck0106369h0003). The website construction and web-based survey were outsourced to the NTT Data Institute of Management Consulting, Inc. The participants received JPY 25 (~US $0.23) and an additional JPY 5 (~US $0.045) if they joined the follow-up survey.

## Results

### Analysis of Participant Demographics

A total of 2175 participants were recruited. Stratifying factors, such as sex and willingness to undergo HPV vaccination prior to randomization, were evenly allocated (Multimedia Appendix 1). The retention rates at the 3-month follow up survey were 89.6% (976/1089) in the intervention group and 76.5% (831/1086) in the control group. The following variables did not demonstrate significant differences between the intervention and control groups: age group, marital status, number of children, number of daughters, education, household income, and tobacco use (Table 1).

**Table 1:** Characteristics and baseline knowledge level of the participants recruited via a website before intervention (March 19-30, 2020).

Characteristics and baseline knowledge		Total (N=2175)	Intervention (N=1089)	Control (N=1086)	*P* value^a^
**Sex, n (%)**					
	Male (father)	1266 (58.2)	633 (58.1)	633 (58.3)	N/A^b^
	Female (mother)	909 (41.8)	456 (41.9)	453 (41.7)	.94
**Age (years), n (%)**					
	30-39	138 (6.3)	76 (7.0)	62 (5.7)	N/A
	40-49	1306 (60.0)	629 (57.8)	677 (62.3)	N/A
	50-59	731 (33.6)	384 (35.3)	347 (32.0)	.08
**Marital status, n (%)^c^**					
	Married	1439 (92.8)	734 (93.1)	705 (92.5)	N/A
	Unmarried	111 (7.2)	54 (6.9)	57 (7.5)	.31
**Number of children, n (%)**					
	1	899 (41.3)	457 (42.0)	442 (40.7)	N/A
	2	1048 (48.2)	521 (47.8)	527 (48.5)	N/A
	3	209 (9.6)	104 (9.6)	105 (9.7)	N/A
	4	19 (0.9)	7 (0.6)	12 (1.1)	.82
**Number of daughters, n (%)**					
	1	1651 (75.9)	818 (75.1)	833 (76.7)	N/A
	2	482 (22.2)	255 (23.4)	227 (20.9)	N/A
	3	38 (1.7)	16 (1.5)	22 (2.0)	N/A
	4	4 (0.2)	0	4 (0.4)	.15
**Education, n (%)^c^**					
	Less than high school graduate	21 (1.4)	7 (0.9)	14 (1.8)	N/A
	High school graduate	355 (22.9)	189 (24.0)	166 (21.8)	N/A
	More than high school graduate	1174 (75.7)	592 (75.1)	582 (76.4)	.203
Household income (million JPY/year)^c,d^, mean (SD)		7.41 (4.68)	7.50 (5.43)	7.32 (3.74)	.44
**Willingness for HPV^e^** **vaccination before randomization, n (%)**					
	Yes	191 (8.8)	97 (8.9)	94 (8.7)	N/A
	No	1984 (91.2)	992 (91.1)	992 (91.3)	.84
**Tobacco use, n (%)**					
	Smoker	563 (25.9)	285 (26.2)	278 (25.6)	N/A
	Nonsmoker	1069 (49.1)	549 (50.4)	520 (47.9)	N/A
	Previous smoker	543 (25.0)	255 (23.4)	288 (26.5)	.24
**Awareness level from AQs^f^ 1-7, n (%)**					
	No awareness	1017 (46.8)	507 (46.6)	510 (47.0)	N/A
	Normal awareness	1158 (53.2)	582 (53.4)	576 (53.0)	.85
**AQ 1 (possibility to find both cancer and precancerous lesions through cervical cancer screening), n (%)**					
	Already known	591 (27.2)	303 (27.8)	288 (26.5)	N/A
	Not known	1584 (72.8)	786 (72.2)	798 (73.5)	.49
**AQ 2 (association of sexual experience with HPV infection), n (%)**					
	Already known	834 (38.3)	412 (37.8)	422 (39.6)	N/A
	Not known	1341 (61.7)	677 (62.2)	644 (60.4)	.62
**AQ 3 (cervical cancer screening necessary for women even after vaccination), n (%)**					
	Already known	387 (17.8)	188 (17.3)	199 (18.3)	N/A
	Not known	1788 (82.2)	901 (82.7)	887 (81.7)	.52
**AQ 4 (effectiveness associated with HPV vaccination), n (%)**					
	Already known	339 (15.6)	164 (15.1)	175 (16.1)	N/A
	Not known	1836 (84.4)	925 (84.9)	911 (83.9)	.498
**AQ 5 (adverse events associated with HPV vaccination), n (%)**					
	Already known	562 (25.8)	269 (24.7)	293 (27.0)	N/A
	Not known	1613 (74.2)	820 (75.3)	793 (73.0)	.23
**AQ 6 (HPV can cause anal cancer and oropharyngeal cancer in males, other than cervical cancer in women), n (%)**					
	Already known	339 (15.6)	164 (15.1)	175 (16.1)	N/A
	Not known	1836 (84.4)	925 (84.9)	911 (83.9)	.498
**AQ 7 (national immunization program of HPV vaccine and suspension of the proactive recommendation from the Japanese government), n (%)**					
	Already known	448 (20.6)	228 (20.9)	220 (20.3)	N/A
	Not known	1727 (79.4)	861 (79.1)	866 (79.7)	.696
**Last Pap^g^ test^h^, n (%)**					
	<2 years	468 (51.5)	235 (51.5)	233 (51.4)	N/A
	2-5 years	95 (10.5)	46 (10.1)	49 (10.8)	N/A
	>5 years	153 (16.8)	77 (16.9)	76 (16.8)	N/A
	Never	179 (19.7)	91 (20.0)	88 (19.4)	N/A
	Unknown	14 (1.5)	7 (1.5)	7 (1.5)	.87
**HPV vaccination^h^, n (%)**					
	Already vaccinated	11 (1.2)	7 (1.5)	4 (0.9)	N/A
	Not yet vaccinated	837 (92.1)	420 (92.1)	417 (92.1)	N/A
	Unknown	61 (6.7)	29 (6.4)	32 (7.1)	.81

^a^*P* values were estimated using chi-square and Student *t* tests.

^b^N/A: not applicable.

^c^Only participants who provided background information about marital status, educational level, and household income (n=1550, 71.3%).

^d^JPY 110=US $1 USD.

^e^HPV: human papillomavirus.

^f^AQ: awareness question.

^g^Pap: Papanicolaou.

^h^Only female participants (n=909, 41.8%).

### Baseline Awareness of HPV and Prevention of Cervical Cancer

For AQ1 to AQ7 on HPV and HPV awareness, there were no significant differences in the recognition rates across all 7 questions between the intervention and control groups (Table 1). Only 339 (15.6%) parents were aware of the effectiveness of HPV vaccination (AQ4), while 562 (25.8%) were aware of the adverse effects (AQ5). The highest awareness rate was seen in the question about the causal relationship between sexual experience and HPV (AQ2; n=834, 38.3%). Among women, there was no significant difference in the pattern of the Pap test and HPV vaccination between the 2 groups.

### Willingness to Vaccinate Daughters and Other Areas of HPV Vaccination Awareness

Only 476 (21.9%) parents displayed a positive attitude toward HPV vaccination for their daughters (WQ 1). Compared to parents in the control group, an additional 7.5% parents responded affirmatively in the intervention group (279/1089 vs 197/1086, 25.6% vs 18.1%; RR 1.41, 95% CI 1.20-1.66); see Table 2. Affirmative attitudes toward other areas, such as undergoing a pap smear (RR 1.14, 95% CI 1.05-1.24), desiring the recommendation from the government (RR 1.36, 95% CI 1.29-1.55), and disseminating HPV vaccination information to someone by social media (RR 1.41, 95% CI 1.20-1.66), were also higher in the intervention group (Table 2).

**Table 2:** Comparison of attitudes and willingness toward HPV^a^ vaccination for the prevention of cervical cancer after intervention from the web survey between the intervention group and the control group (March 19-30, 2020).

Responses to WQs^b^		Total (N=2175), n (%)		Intervention (N=1089), n (%)	Control (N=1086), n (%)		Yes vs other	
						RR^c^ (95% CI)		*P* value^d^
**WQ 1. Would you consider getting your daughter vaccinated against HPV?**								
	Yes	476 (21.9)	279 (25.6)		197 (18.1)	1.41 (1.20-1.66)		<.001
	No	500 (23.0)	200 (18.4)		300 (27.6)	N/A^e^		N/A
	I’m not sure	1199 (55.1)	610 (56.0)		589 (54.2)	N/A		N/A
**WQ 2. Would you consider undergoing a Pap^f^ smear? If male, would you want your family member or partner to undergo a smear test?**								
	Yes	1066 (49.0)	569 (52.2)		497 (45.8)	1.14 (1.05-1.24)		.003
	No	357 (16.4)	153 (14.0)		204 (18.8)	N/A		N/A
	I’m not sure	752 (34.6)	367 (33.7)		385 (35.5)	N/A		N/A
**WQ 3. Do you think the HPV vaccination program should be actively recommended by the government?**								
	Yes	626 (28.8)	361 (33.1)		265 (24.4)	1.36 (1.29-1.55)		<.001
	No	371 (17.1)	156 (14.3)		215 (19.8)	N/A		N/A
	I’m not sure	1178 (54.2)	572 (52.5)		606 (55.8)	N/A		N/A
**WQ 4. Do you plan to inform family members, friends, or others about cervical cancer prevention and screening (through Instagram, Facebook, LINE, Twitter, TikTok, etc)?**								
	Yes	481 (22.1)	282 (25.9)		199 (18.3)	1.41 (1.20-1.66)		<.001
	No	797 (36.6)	357 (32.8)		440 (40.5)	N/A		N/A
	I’m not sure	897 (41.2)	450 (41.3)		447 (41.2)	N/A		N/A
**WQ 5. Are you going to make an appointment for HPV vaccination for your daughter as soon as possible?**								
	Yes	497 (22.9)	298 (27.4)		199 (18.3)	1.49 (1.27-1.75)		<.001
	No	1678 (77.1)	791 (72.6)		887 (81.7)	N/A		N/A

^a^HPV: human papillomavirus.

^b^WQ: willingness question.

^c^RR: risk ratio.

^d^*P* values were estimated using the chi-square test. If Bonferroni correction was applied, the threshold of significance level was adjusted to .05/5=.01, which showed the *P* values in this table were still significantly low.

^e^N/A: not applicable.

^f^Pap: Papanicolaou.

### Sex-wise Attitudes Toward HPV Vaccination and Awareness Regarding the Prevention of Cervical Cancer

The comparison of attitudes toward HPV vaccination and awareness regarding the prevention of cervical cancer according to sex are shown in Table 3.

Differences between sexes were identified in all questions. Fathers were more likely to have an affirmative attitude toward HPV vaccination for their daughters in the intervention group (fathers: RR 1.50, 95% CI 1.25-1.81; mothers: RR 1.21, 95% CI 0.88-1.66). Additionally, fathers in the intervention group were more likely to have affirmative attitudes regarding other areas of awareness, such as undergoing a Pap smear, desiring the recommendation from the government, and disseminating HPV vaccination information to someone by social media (Table 3). In an overall comparison between fathers and mothers irrespective of the short-film intervention, the willingness to consider HPV vaccination for daughters was significantly higher in fathers than in mothers (n=343, 27.1%, vs n=133, 14.6%, *P*<.001).

In addition, 650 (71.5%) of 909 mothers knew at least 1 of the items regarding HPV vaccination, while only 508 (40.1%) of 1266 fathers were aware of at least 1 item (Multimedia Appendix 2). However, in the subgroup analysis by awareness level, the intervention increased the willingness to consider HPV vaccination for their daughters in both awareness level groups: 6.0% increase (RR 1.27, 95% CI 1.04-1.55) in the normal-awareness group and 9.2% increase (RR 1.69, 95% CI 1.28-2.22) in the no-awareness group (data not shown).

**Table 3:** Sex-wise comparison of attitudes and willingness toward HPV^a^ vaccination for the prevention of cervical cancer after intervention from the web survey (March 19-30, 2020).

Responses to WQs^b^		Mothers (N=909)						Fathers (N=1266)				
		Total, n (%)	Intervention (N=456), n (%)	Control (N=453), n (%)	Yes vs other RR^c^ (95% CI)	Yes vs other *P* value^d^	Total, n (%)		Intervention (N=633), n (%)	Control (N=633), n (%)	Yes vs other RR^c^ (95% CI)	Yes vs other *P* value^d^
**WQ 1. Would you consider getting your daughter vaccinated against HPV?**												
	Yes	133 (14.6)	73 (16.0)	60 (13.2)	1.21 (0.88-1.66)	.24	343 (27.1)		206 (32.5)	137 (21.6)	1.50 (1.25-1.81)	<.001
	No	286 (31.5)	110 (24.1)	176 (38.9)	N/A^e^	N/A	214 (16.9)		90 (14.2)	124 (19.6)	N/A	N/A
	I'm not sure	490 (53.9)	273 (59.9)	217 (47.9)	N/A	N/A	709 (56.0)		337 (53.2)	372 (58.8)	N/A	N/A
**WQ 2. Would you consider undergoing a Pap^f^ smear? If male, would you want your family member or partner to undergo a smear test?**												
	Yes	523 (57.5)	262 (57.5)	261 (57.6)	1.00 (0.89-1.12)	.96	543 (42.9)		307 (48.5)	236 (37.3)	1.30 (1.14-1.48)	<.001
	No	160 (17.6)	73 (16.0)	87 (19.2)	N/A	N/A	197 (15.6)		80 (12.6)	117 (18.5)	N/A	N/A
	I'm not sure	226 (24.9)	121 (26.5)	105 (23.2)	N/A	N/A	526 (41.5)		246 (38.9)	280 (44.2)	N/A	N/A
**WQ 3. Do you think the HPV vaccination program should be actively recommended by the government?**												
	Yes	167 (18.4)	93 (20.4)	74 (16.3)	1.25 (0.95-1.65)	.114	459 (36.3)		268 (42.3)	191 (30.2)	1.40 (1.21-1.63)	<.001
	No	200 (22.0)	81 (17.8)	119 (26.3)	N/A	N/A	171 (13.5)		75 (11.8)	96 (15.2)	N/A	N/A
	I'm not sure	542 (59.6)	282 (61.8)	260 (57.4)	N/A	N/A	636 (50.2)		290 (45.8)	346 (54.7)	N/A	N/A
**WQ 4. Do you plan to inform family members, friends, or others about cervical cancer prevention and screening (through Instagram, Facebook, LINE, Twitter, TikTok, etc)?**												
	Yes	213 (23.4)	119 (26.1)	94 (20.8)	1.26 (0.99-1.59)	.06	268 (21.2)		163 (25.8)	105 (16.6)	1.55 (1.25-1.93)	<.001
	No	349 (38.4)	148 (32.5)	201 (44.4)	N/A	N/A	448 (35.4)		209 (33.0)	239 (37.8)	N/A	N/A
	I'm not sure	347 (38.2)	189 (41.5)	158 (34.9)	N/A	N/A	550 (43.4)		261 (41.2)	289 (45.7)	N/A	N/A
**WQ 5. Are you going to make an appointment for HPV vaccination for your daughter as soon as possible?**												
	Yes	147 (16.2)	83 (18.2)	64 (14.1)	1.29 (0.96-1.74)	.095	350 (27.6)		215 (34.0)	135 (21.3)	1.59 (1.32-1.92)	<.001
	No	762 (83.8)	373 (81.8)	389 (85.9)	N/A	N/A	916 (72.4)		418 (66.0)	498 (78.7)	N/A	N/A

^a^HPV: human papillomavirus.

^b^WQ: willingness question.

^c^RR: risk ratio.

^d^*P* values were estimated using the chi-square test. If Bonferroni correction was applied, the threshold of significance level was adjusted to .05/10=.005, which showed the *P* values in this table were still significantly low.

^e^N/A: not applicable.

^f^Pap: Papanicolaou.

### Follow-Up Survey After 3 Months

At the follow-up survey after 3 months, 368 (16.9%) parents did not answer the online survey (Figure 1). The remaining 1807 (83.1%) parents were provided with a follow-up questionnaire. The results of the follow-up survey are presented in Table 4. A total of 149 (8.2%) parents responded that their daughters were vaccinated in the past 3 months. However, there was no difference in the vaccination rate in both groups: 77 (7.9%) in the intervention group versus 72 (8.7%) in the control group (RR 0.88, 95% CI 0.66-1.18). Among those who answered that they did not let their daughters get vaccinated (n=1638, 75.3%), only 124 (7.6%) of the parents displayed a positive attitude toward HPV vaccination for their daughters (FQ 1’). Regarding this question, the effect of the short film on willingness to consider HPV vaccination was not observed (8.0% in the intervention group vs 7.1% in the control group, *P*=0.497). We could not find any effect of the intervention on the subsequent 4 questions (FQs 2-5), although the effect was seen in the first survey.

**Table 4:** Comparison of attitudes, willingness, and behaviors toward HPV^a^ vaccination for the prevention of cervical cancer at the 3-month follow-up from the web survey between the intervention group and the control group (June 26-July 6, 2020).

Responses to FQs^b^		Total (N=1807), n (%)		Intervention (N=976), n (%)	Control (N=831), n (%)		Yes vs other	
						RR^c^ (95% CI)		*P* value^d^
**FQ 1. Has your daughter been vaccinated after the first-round questionnaire?**								
	Vaccinated	149 (8.2)	77 (7.9)		72 (8.7)	0.88 (0.66-1.18)		.39
	Only an appointment	20 (1.1)	9 (0.9)		11 (1.3)	N/A^e^		N/A
	Nothing	1638 (90.6)	890 (91.2)		748 (90.0)	N/A		N/A
**FQ 1'. Would you consider getting your daughter vaccinated against HPV?^f^**								
	Yes	124 (7.6)	71 (8.0)		53 (7.1)	1.13 (0.80-1.59)		.497
	No	424 (25.9)	228 (25.6)		196 (26.2)	N/A		N/A
	I’m not sure	1090 (66.5)	591 (66.4)		499 (66.7)	N/A		N/A
**FQ 2. Would you consider undergoing a Pap^g^ smear? If male, would you want your family member or partner to undergo a smear test?**								
	Yes	852 (47.1)	466 (47.7)		386 (46.5)	1.03 (0.93-1.13)		.582
	No	327 (18.1)	163 (16.7)		164 (19.7)	N/A		N/A
	I’m not sure	628 (34.8)	347 (35.6)		281 (33.8)	N/A		N/A
**FQ 3. Do you think the HPV vaccination program should be actively recommended by the government?**								
	Yes	362 (20.0)	196 (20.1)		166 (20.0)	1.01 (0.84-1.21)		.96
	No	362 (20.0)	193 (19.8)		169 (20.3)	N/A		N/A
	I’m not sure	1083 (59.9)	587 (60.1)		496 (59.7)	N/A		N/A
**FQ 4. Do you plan to inform family members, friends, or others about cervical cancer prevention and screening (through Instagram, Facebook, LINE, Twitter, TikTok, etc)?**								
	Yes	314 (17.4)	174 (17.8)		140 (16.8)	1.06 (0.86-1.30)		.583
	No	743 (41.1)	389 (39.9)		354 (42.6)	N/A		N/A
	I’m not sure	750 (41.5)	413 (42.3)		337 (40.6)	N/A		N/A
**FQ 5. Are you going to make an appointment for HPV vaccination for your daughter as soon as possible?^f^**								
	Yes	204 (12.5)	111 (12.5)		93 (12.4)	1.00 (0.78-1.30)		.98
	No	1434 (87.5)	779 (87.5)		655 (87.6)	N/A		N/A

^a^HPV: human papillomavirus.

^b^FQ: follow-up question.

^c^RR: risk ratio.

^d^*P* values were estimated using the chi-square test.

^e^N/A: not applicable.

^f^Asked only to participants who answered no in FQ 1.

^g^Pap: Papanicolaou.

## Discussion

### Principal Findings

This web-based RCT showed that showing a 4-minute-long film on the story of a patient with cervical cancer increases parents’ willingness to consider HPV vaccination for their daughters. Furthermore, the willingness to undergo cervical cancer–screening tests and to disseminate HPV vaccination–related information about what they saw and felt also increased in the intervention group. These effects were observed in the fathers’ cohort but not in the mothers’ cohort. This result was similar to our prior RCT, which showed that a brief educational tool using the importance of HPV vaccination increases the willingness to consider the vaccination for their daughters and sons [4]. The noteworthy point is that both brief material based on medical evidence and a short film based on a cervical cancer survivor’s story positively affect only men. In contrast, the story of a cancer patient did not change the parents’ behavior toward HPV vaccination, and 3 months later, the parents' willingness toward vaccination was not sustained. Even the mothers’ personal experiences of cervical cancer were not a sufficient factor in increasing their children’s vaccination rate [15], so it seems cogent that a cancer survivor’s story does not impact parents’ behavior regarding their daughters’ vaccination against HPV.

Similar to the prior RCT, a possible reason the educational intervention was more effective among men could be that women had more existing awareness about HPV and HPV vaccination and had more negative sentiments against HPV vaccination (Multimedia Appendix 2). In fact, there was a significant difference between fathers and mothers in the awareness level of HPV vaccination. However, we found that the intervention increased the willingness to consider HPV vaccination for their daughters irrespective of awareness level. Thus, our trial indicated that sex differences may be a significant factor in the decision-making of HPV vaccination for daughters. Although several studies have described the mothers’ hesitancy to get their children vaccinated against COVID-19 as a potential factor for a lower vaccination rate [16], little is known about parents’ sex differences affecting the likelihood of HPV vaccination for their children. Even if the awareness level is not a factor influencing their willingness, there might be a difference between fathers and mothers regarding anxiety or the decision-making process. We need to consider measuring this sort of index to examine possible factors affecting the gender difference toward HPV vaccination.

In the follow-up survey, we did not find a significant intervention effect on HPV vaccination for the participants’ daughters. There may be some reason for this. First, this awareness change happened to just 1 parent. The decision-making process with another parent or daughter would be needed to take an action for vaccination. Therefore, this type of indirect intervention could have some limitations in its impact. Surprisingly, a total of 8.2% of the participants had their daughters vaccinated after participation in this trial, which was higher than the general HPV vaccination rate in Japan at the time of the study [5,6]. The information we provided, including that given prior to randomization and awareness questions, might have changed their attitudes and behaviors. The information including general facts addressing safety concerns and the benefits regarding HPV vaccination could reduce the participants’ hesitancy [17]. Moreover, the effect of intervention was not present anymore 3 months later, implying that this particular intervention may be limited in sustaining one’s attitudes toward HPV vaccination over a period of time. We did not limit the participants’ access to any resources, such as health care providers and health information from various media. This possibly reduced the effect of the intervention.

Although face-to-face educational approaches with parents might be an effective way to improve awareness and understanding of the vaccination [18], we need to increasingly utilize the online approach according to the rapid growth in the share of social media users worldwide [19]. Abundant information regarding health issues is provided and disseminated mainly through social media [20]. Furthermore, information based on public health facts and misinformation based on no-scientific theory, provaccine posts, and antivaccine posts is mixed [21-23]. The problem is that antivaccine information has a tendency to be created by individuals who do not have a medical background, while the information still sounds plausible for many people [19]. In some countries, including Japan, some European countries, and the Unites States, nationwide vaccine hesitancy in regard to HPV vaccination was seen to be caused by online and offline dissemination of misleading beliefs [2,4,5,24-27]. Although many researchers know that 1 of the main causes of vaccine hesitancy may be information overload and misinformation, which is also called an “infodemic” [28,29], medical professionals should also provide information about HPV vaccination to the public as a reliable information resource and keep encouraging parents to get their children vaccinated [27]. Additionally, a national scale approach that is coordinated among health care providers, parents, media, and policy makers should be utilized to combat disinformation and misinformation regarding vaccines [24,30].

Twitter [23,31,32], Instagram [21], and Facebook [32-34] have been proposed as promising educational methods in the past 5 years; however, little is still known about the best specific way to mitigate vaccine hesitancy for HPV vaccination [35-37]. A study reported that using social media to promote health behavior leads to a significant improvement in behavioral change [38]. For instance, in the United States, the use of social media has grown in the past decade [39]; therefore, it might be a great platform to educate people about HPV vaccination–related information. The data also shows the rate of use by age and sex [39]. A strategy by age or sex based on the theory of social marketing [2,40] will be valuable if we proceed with a campaign of HPV vaccination based on the results of studies like ours [4], although social media could lead to parents’ vaccine hesitancy [16,30]. As a systematic review suggested, the identified strategy should be carefully tailored according to specific populations [37], considering the pros and cons of social media.

### Limitations

Although this study displayed a promising effect on overcoming vaccine hesitancy, we need to consider several limitations. First, the cohort of the specified internet survey population used in this study may have some selection bias. The cohort has a higher educational background and is wealthier than the general Japanese population; approximately JPY 5.16 million is the average household income [41]. Additionally, the gender imbalance in this study’s population needs to be considered as another selection bias.

Second, interventions using this kind of awareness material are usually not universal, that is, the effect may vary when other materials are used. In particular, the effect might change depending on the individuals in the film, the content of the anecdotes, and the length of the film.

Third, although this trial was designed to assess the sustainability of awareness change and concrete behavior for HPV vaccination, we could not incorporate the actual vaccination records into our research. Therefore, the reported vaccination rate might be different from the actual vaccination rate.

Fourth, there was some potential social desirability bias that affected the effectiveness of the intervention at the first survey; on the contrary, the effect could not be seen in the follow-up survey. We have to take a look at the effectiveness of the film, considering the existence of a bias away from null.

Given that this trial implemented under the suspension of proactive recommendation by the Japanese government, we have a tentative plan to conduct the same trial after resuming the national vaccination program. In addition, there were potential negative impacts in the study period, which was carried out during the COVID-19 pandemic. A lockdown was not declared in Japan during the study period; however, a portion of participants might have refrained from going to the hospital for nonurgent vaccination.

### Conclusion

#### Inference of the Study Findings

This study demonstrated a positive immediate effect on the willingness for HPV vaccination in parents who have daughters, following intervention using a short film on cervical cancer, especially among fathers. Such an approach is promising for overcoming the hesitancy toward HPV vaccination. Additionally, this RCT showed the importance of the father’s role in improving the HPV vaccination rate and overcoming vaccine hesitancy.

#### Impact of the Findings

An anecdotal cervical cancer survivor’s story increases the willingness of Japanese parents to consider HPV vaccination for their daughters. However, this type of intervention might not sustain their motivation months afterward.

## Appendix

Multimedia Appendix 1 Flow diagram of the study.

Multimedia Appendix 2 Characteristics and knowledge level between father and mother.

Multimedia Appendix 3 CONSORT-eHEALTH checklist (V 1.6.1).

## Abbreviations

AQ: awareness question
FQ: follow-up question
HPV: human papillomavirus
MHLW: Ministry of Health, Labour and Welfare
Pap: Papanicolaou
RCT: randomized controlled trial
RR: risk ratio
WQ: willingness question
