# Supplementary material for: The Effect of a Web-Based Cervical Cancer Survivor’s Story on Parents' Behavior and Willingness to Consider Human Papillomavirus Vaccination for Daughters: Randomized Controlled Trial
Source: JMIR Public Health Surveill. 2022 May 25;8(5):e34715. doi: 10.2196/34715 (PMC9178460; doi:10.2196/34715)
Supplement: Multimedia Appendix 1 [file publichealth_v8i5e34715_app1.pptx]

## Slide 1
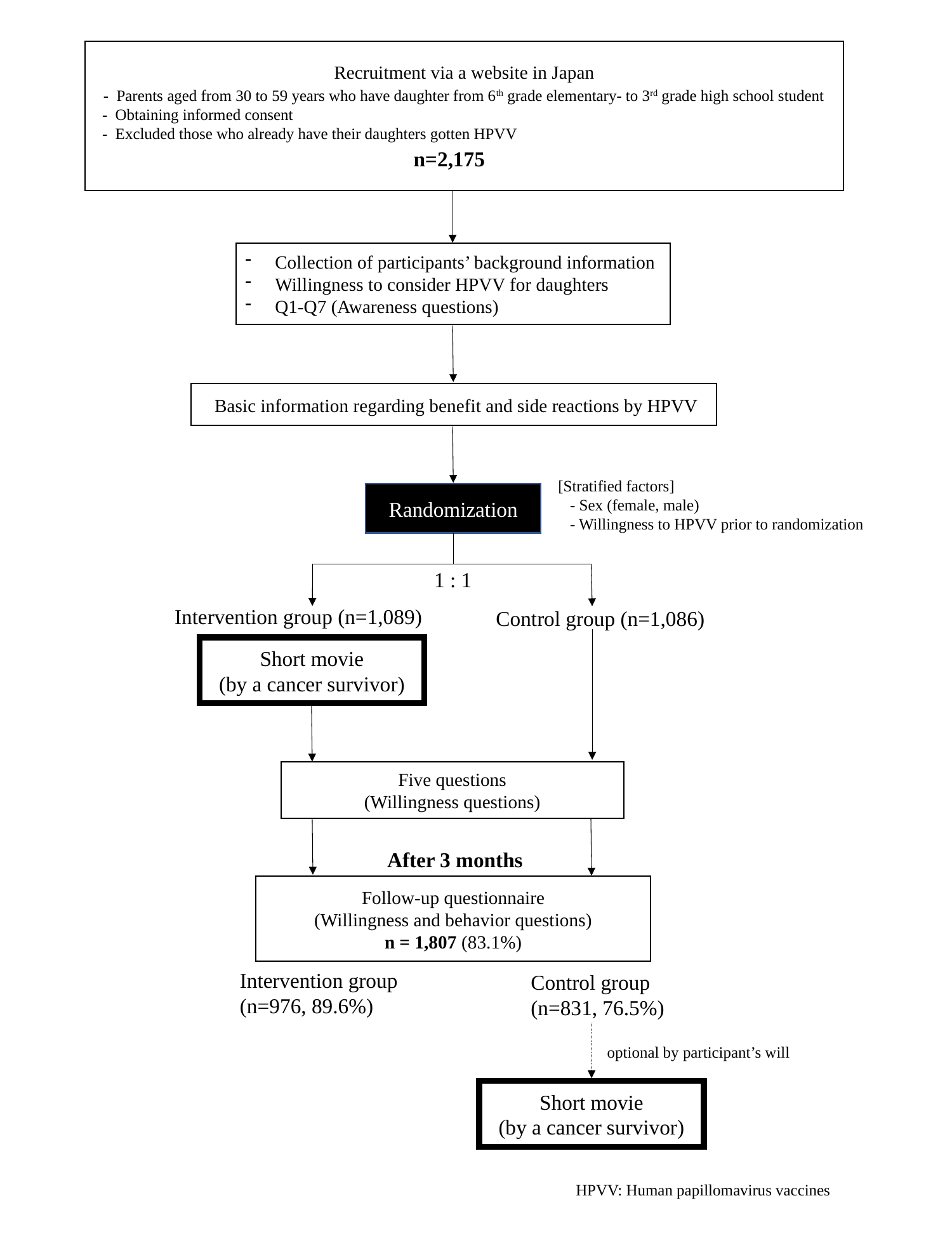

Recruitment via a website in Japan
 - Parents aged from 30 to 59 years who have daughter from 6th grade elementary- to 3rd grade high school student
 - Obtaining informed consent
 - Excluded those who already have their daughters gotten HPVV
 n=2,175
Collection of participants’ background information
Willingness to consider HPVV for daughters
Q1-Q7 (Awareness questions)
 Basic information regarding benefit and side reactions by HPVV
 [Stratified factors]
 - Sex (female, male)
 - Willingness to HPVV prior to randomization
Randomization
1 : 1
Intervention group (n=1,089)
Control group (n=1,086)
Short movie
(by a cancer survivor)
Five questions
(Willingness questions)
After 3 months
Follow-up questionnaire
(Willingness and behavior questions)
n = 1,807 (83.1%)
Intervention group
(n=976, 89.6%)
Control group
(n=831, 76.5%)
optional by participant’s will
Short movie
(by a cancer survivor)
HPVV: Human papillomavirus vaccines
