# Supplementary material for: The Effect of a Web-Based Cervical Cancer Survivor’s Story on Parents' Behavior and Willingness to Consider Human Papillomavirus Vaccination for Daughters: Randomized Controlled Trial
Source: JMIR Public Health Surveill. 2022 May 25;8(5):e34715. doi: 10.2196/34715 (PMC9178460; doi:10.2196/34715)
Supplement: Multimedia Appendix 2 [file publichealth_v8i5e34715_app2.docx]

| **Multimedia Appendix 1. Characteristics and knowledge level between father and mother** | | | |  |  |  |  |
| --- | --- | --- | --- | --- | --- | --- | --- |
|  | | | | | | | |
| Characteristic | All | | Men (Father) | | Women (Mother) | | *p* value ^a^ |
|  | (n = 2,175) | | (n = 1,266) | | (n = 909) | |  |
|  | n | % | n | % | n | % |  |
| Randomization |  |  |  |  |  |  |  |
| Intervention | 1,089 | 50.1 | 633 | 50.0 | 456 | 50.2 |  |
| Control | 1,086 | 49.9 | 633 | 50.0 | 453 | 49.8 | .939 |
| Age |  |  |  |  |  |  |  |
| 30–39 | 138 | 6.3 | 54 | 4.3 | 84 | 9.2 |  |
| 40–49 | 1306 | 60.0 | 675 | 53.3 | 631 | 69.4 |  |
| 50–59 | 731 | 33.6 | 537 | 42.4 | 194 | 21.3 | <.001 |
| Marital status, n (%)^b^ |  |  |  |  |  |  |  |
| Married | 1439 | 92.8 | 825 | 92.8 | 614 | 92.9 |  |
| Unmarried | 111 | 7.2 | 64 | 7.2 | 47 | 7.1 | .907 |
| The number of daughter ^b^, n (%) |  |  |  |  |  |  |  |
| 1 | 1651 | 75.9 | 949 | 75.0 | 702 | 77.2 |  |
| 2 | 482 | 22.2 | 287 | 22.7 | 195 | 21.5 |  |
| 3 | 38 | 1.7 | 27 | 2.1 | 11 | 1.2 |  |
| 4 | 4 | 0.2 | 3 | 0.2 | 1 | 0.1 | .393 |
| Education, n (%) ^b^ |  |  |  |  |  |  |  |
| Less than high school graduate | 21 | 1.4 | 11 | 1.2 | 10 | 1.5 |  |
| High school graduate | 355 | 22.9 | 201 | 22.6 | 154 | 23.3 |  |
| More than high school graduate | 1174 | 75.7 | 1054 | 76.2 | 745 | 75.2 | <.001 |
|  |  |  |  |  |  |  |  |
| Household income ^b^ (million JPY ^c^/year), mean (SD ^d^) | 7.41 (4.68) | | 7.54 (4.29) | | 7.23 (5.15) | | .197 |
|  |  |  |  |  |  |  |  |
| Willingness to HPVV^e^ before randomization, n (%) |  |  |  |  |  |  |  |
| Yes | 191 | 8.8 | 140 | 11.1 | 51 | 5.6 |  |
| No | 1984 | 91.2 | 1126 | 88.9 | 858 | 94.4 | <.001 |
|  |  |  |  |  |  |  |  |
| Tobacco use |  |  |  |  |  |  |  |
| Smoker | 563 | 25.9 | 453 | 35.8 | 110 | 12.1 |  |
| Non-smoker | 1,069 | 49.1 | 398 | 31.4 | 145 | 16.0 |  |
| Previous smoker | 543 | 25.0 | 415 | 32.8 | 654 | 71.9 | <.001 |
|  |  |  |  |  |  |  |  |
| Awareness level from AQ1-7 |  |  |  |  |  |  |  |
| No-awareness | 1,017 | 46.8 | 758 | 59.9 | 259 | 28.5 |  |
| Normal-awareness | 1,158 | 53.2 | 508 | 40.1 | 650 | 71.5 | <.001 |
|  |  |  |  |  |  |  |  |
| AQ1 It is possible to find both cancer and pre-cancerous lesions through cervical cancer screening | | | | | | | |
| Already known | 591 | 27.2 | 223 | 17.6 | 368 | 40.48 |  |
| Not known | 1,584 | 72.8 | 1043 | 82.4 | 541 | 59.52 | <.001 |
| AQ2 Sexual experience is associated with HPV infection | | | | | | | |
| Already known | 834 | 38.3 | 392 | 31.0 | 442 | 48.6 |  |
| Not known | 1,341 | 61.7 | 874 | 69.0 | 467 | 51.4 | <.001 |
| AQ3 Cervical cancer screening is necessary for women even after vaccinated | | | | | | | |
| Already known | 387 | 17.8 | 141 | 11.1 | 246 | 27.1 |  |
| Not known | 1,788 | 82.2 | 1125 | 88.9 | 663 | 72.9 | <.001 |
| AQ4 Effectiveness associated with HPV vaccination | | | | | | | |
| Already known | 339 | 15.6 | 152 | 12.0 | 187 | 20.6 |  |
| Not known | 1,836 | 84.4 | 1114 | 88.0 | 722 | 79.4 | <.001 |
| AQ5 Adverse events associated with HPV vaccination | | | | | | | |
| Already known | 562 | 25.8 | 172 | 13.6 | 390 | 42.9 |  |
| Not known | 1,613 | 74.2 | 1094 | 86.4 | 519 | 57.1 | <.001 |
| AQ6 HPV causes anal cancer and pharyngeal cancer regardless of sex | | | | | | | |
| Already known | 339 | 15.6 | 74 | 5.8 | 59 | 6.5 |  |
| Not known | 1,836 | 84.4 | 1192 | 94.2 | 850 | 93.5 | .535 |
| AQ7 The position of HPV vaccine in japanese law and the current thoughts of Japansese government | | | | | | | |
| Already known | 448 | 20.6 | 122 | 9.6 | 326 | 35.9 |  |
| Not known | 1,727 | 79.4 | 1144 | 90.4 | 583 | 64.1 | <.001 |

| a *P* value are estimiated using the chi-square and Student *t* tests. |
| --- |
| b Only participants who answered the background information (n=1550). |
| c 1 USD = 110 JPY |
| d SD, Standard deviation. |
| e HPVV: Human papillomavirus Vaccination |
